# SNP identification, verification, and utility for population genetics in a non-model genus

**DOI:** 10.1186/1471-2156-11-32

**Published:** 2010-04-30

**Authors:** Larissa M Williams, Xin Ma, Adam R Boyko, Carlos D Bustamante, Marjorie F Oleksiak

**Affiliations:** 1 Department of Environmental and Molecular Toxicology, Box 7633, North Carolina State University, Raleigh, NC 27695-7633, USA; 2 Department of Biological Statistics and Computational Biology, Cornell University, Ithaca, NY 14853, USA; 3 Rosenstiel School of Marine and Atmospheric Sciences, University of Miami, 4600 Rickenbacker Causeway, Miami, FL 33149, USA

## Abstract

**Background:**

By targeting SNPs contained in both coding and non-coding areas of the genome, we are able to identify genetic differences and characterize genome-wide patterns of variation among individuals, populations and species. We investigated the utility of 454 sequencing and MassARRAY genotyping for population genetics in natural populations of the teleost, *Fundulus heteroclitus *as well as closely related *Fundulus *species (*F. grandis*, *F. majalis *and *F. similis*).

**Results:**

We used 454 pyrosequencing and MassARRAY genotyping technology to identify and type 458 genome-wide SNPs and determine genetic differentiation within and between populations and species of *Fundulus*. Specifically, pyrosequencing identified 96 putative SNPs across coding and non-coding regions of the *F. heteroclitus *genome: 88.8% were verified as true SNPs with MassARRAY. Additionally, putative SNPs identified in *F. heteroclitus *EST sequences were verified in most (86.5%) *F. heteroclitus *individuals; fewer were genotyped in *F. grandis *(74.4%), *F. majalis *(72.9%), and *F. similis *(60.7%) individuals. SNPs were polymorphic and showed latitudinal clinal variation separating northern and southern populations and established isolation by distance in *F. heteroclitus *populations. In *F. grandis*, SNPs were less polymorphic but still established isolation by distance. Markers differentiated species and populations.

**Conclusions:**

In total, these approaches were used to quickly determine differences within the *Fundulus *genome and provide markers for population genetic studies.

## Background

High throughput sequencing and genotyping has become increasingly faster, less expensive and more accurate. In recent years this has lead to the establishment of myriad data sets ranging from increased coverage of variation in the human genome at the individual level [1-5] to the sequencing of non-model prokaryotic and eukaryotic genomes and transcriptomes [6-11]. For many organisms sequencing of entire genomes is still unattained, but smaller, more targeted portions of the genome can be easily sequenced and genotyped. Such data can provide genome-wide sequence information which can be used to characterize population and selection pressure parameters as well as provide evolutionary insights that are broadly applicable [12].

One non-model genus, *Fundulus*, includes closely related species that range in physiology, environmental and habitat preference, and geographic locales; *Fundulus heteroclitus *and *Fundulus majalis *inhabit the Atlantic coast, and *Fundulus grandis *and *Fundulus similis *inhabit the Gulf Coast. Many *Fundulus *species and/or populations have extensive euryhaline capabilities, respond well to varying ranges of hypoxia [13-15], live along a steep thermocline, and have adapted to extremely polluted areas [16]. A variety of studies have investigated the underlying genetic basis of this teleosts' phenotypic plasticity. While some of the transcriptome is known for *F. heteroclitus *[17-27] much of the genome-wide variation within and between populations and species for this genus is relatively unknown.

Establishing a set of genetic markers, which can be used to assess regions of the genome involved in local adaptation and in speciation is important to understand fundamental similarities and differences between populations and species of *Fundulus*. Once markers are established they can be further studied to look for signatures of selection to any number of evolutionary forces (*e.g*., pollution, hypoxia, salinity, temperature). A few studies have established genetic differences between populations of *F. heteroclitus *mainly with respect to phylogeographic constraints [28,29] or selection [30-38]. These studies used microsatellite, mitochondrial DNA, and AFLP analyses as well as targeted gene approaches. Single nucleotide polymorphisms (SNPs) are a useful starting point to scan large and disparate regions of the genome due to their abundance in both coding and non-coding regions, their co-dominant nature, and lack of ambiguity.

SNPs have been used to establish differences between individuals [39], populations [40-42] and species [43,44]. They also are useful markers for propensity to disease [45-47], disease states [48], and evidence of the genetic basis of adaptation [49-52]. In vertebrates, a SNP occurs on average every 100 to 1000 base pairs and often is in linkage disequilibrium with many other SNPs along the chromosome, forming strong haplotypes, which can be easily identified [53]. Unfortunately, SNP resources are not readily available in the majority of non-model species lacking genomic resources. With this in mind, we set out to establish a set of SNP markers to identify differences between *Fundulus *populations and species.

## Methods

### Sample Collection and Extraction

**Figure 1:**
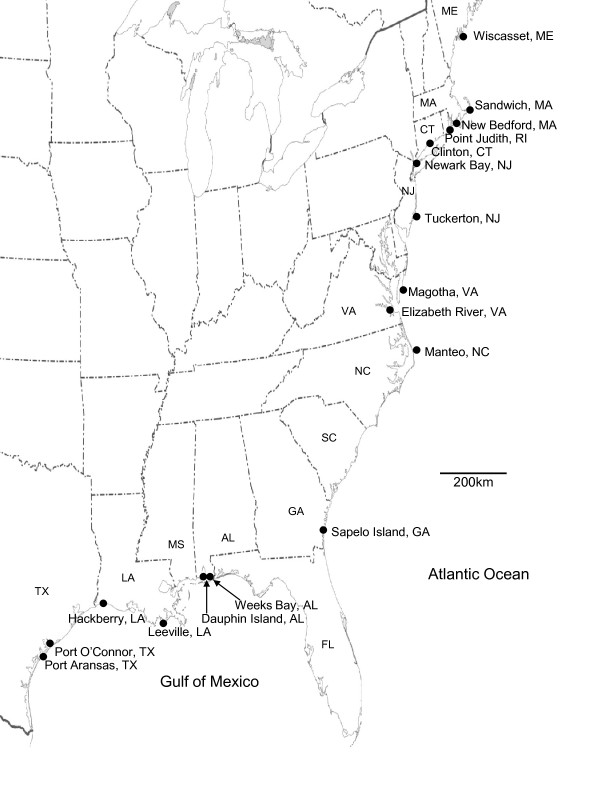
**Sampling sites for *Fundulus *species**. *F. heteroclitus *was collected along the east coast of the United States and *F. grandis *was collected along the Gulf of Mexico coast.

*F. heteroclitus *were collected using minnow traps during the spring of 2005. Spleen and testes were sampled from 20 individuals from each of ten collection sites along the East coast of the United States (Figure 1). *F. grandis *were collected using minnow traps during the winter of 2009 (Figure 1). Fin clips were sampled from 15 individuals from each of the six collection sites along the Gulf Coast of the United States. Spleen from *F. majalis *was extracted from 13 individuals from Woods Hole, Massachusetts and 10 individuals from Sapelo Island, GA. Spleen also was extracted from *F. similis *collected from Pensacola, Florida (3 individuals) and Corpus Christi, Texas (8 individuals).

Genomic DNA from spleen and testes was extracted by phenol and chloroform as described in Wirgin *et al*. [54], and DNA was resuspended in 50 μL 0.1× TE buffer. Genomic DNAs from fin clips were extracted using a modified version of Aljanabi and Martinez [55] and DNA was resuspended in 50 μL 0.1× TE buffer. This experiment was performed according to an approved Institutional Animal Care and Use Committee at North Carolina State University.

### DNA Pyrosequencing

*F. heteroclitus *genomic DNAs (500 ng) from eight individuals in each of ten collection sites (all sites except Point Judith, RI, Figure 1A) were digested individually with 1 U BspE1 (New England Biolabs, MA) and 1 U EcoRI (New England Biolabs, MA). Samples were incubated for three hours at 37°C in a total volume of 30 μL containing Buffer 3 (New England Biolabs, MA). Adaptors (Table 1) to each of the restriction sites, 25 mM ATP, and 1 U of T4 DNA ligase (Epicentre) were added to reactions and incubated at 16°C overnight. A 2' O-methyl block was added to the 3' cytosine base on the adapter. This block assured that only those fragments digested with both BspEI and EcoRI would be amplified with PCR and prevented amplification of fragments with the same type of restriction site on both ends of the fragment.

**Table 1:** Adapters and primers used in the amplification of genomic DNA.

Adapters	
	
**BspEI (5' to 3')**	

GACGATGAGTCCTGAGC	
CTGCTACTCTCAGGACTCGGGCC	
	
**EcoRI (5' to 3')**	

CTAGAGTCCTAGTAGCACCTCGTAGACTGCGTACC *CATCTGACGCATGGTTAA	
	
**Preselective Primers**	

	
**EcoRI (5' to 3')**	

CTGAGTCCTAGTAGCACC	
	
**BspEI (5' to 3')**	

GACGATGAGTCCTGAGC	
	
**Selective Primers**	

	
**EcoRI (5' to 3')**	

GACTGCGTACCAATTCAAG	
	
**BspEI (5' to 3')**	

GACGATGAGTCCTGAGCC	
	
**Barcoded Primers**	

	
**EcoRI (5' to 3')**	

1	GCCTCCCTCGCGCCATCAGAGCCTAAGCTGACTGCGTACCAATTCAAG
2	GCCTCCCTCGCGCCATCAGAGTTCAAGTCGACTGCGTACCAATTCAAG
3	GCCTCCCTCGCGCCATCAGACTTGAACTGGACTGCGTACCAATTCAAG
4	GCCTCCCTCGCGCCATCAGACGGTAACGTGACTGCGTACCAATTCAAG
5	GCCTCCCTCGCGCCATCAGATCCGAATCGGACTGCGTACCAATTCAAG
6	GCCTCCCTCGCGCCATCAGATGGCAATGCGACTGCGTACCAATTCAAG
7	GCCTCCCTCGCGCCATCAGCAGGTCCAGTGACTGCGTACCAATTCAAG
8	GCCTCCCTCGCGCCATCAGCATTGCCATGGACTGCGTACCAATTCAAG
9	GCCTCCCTCGCGCCATCAGCTAAGCCTAGGACTGCGTACCAATTCAAG
10	GCCTCCCTCGCGCCATCAGCGAATCCGATGACTGCGTACCAATTCAAG
	
**BspEI (5' to 3')**	

	
GCCTTGCCAGCCCGCTCAGGACGATGAGTCCTGAGCC	

*Star indicates location of 2' O-methyl block.

Preselective PCR reactions with primers specific to adaptors (Table 1) were performed in a total volume of 25 μL containing 2 μL of diluted (1:10 in 0.1× Tris-EDTA buffer) ligation product with EcoRI primer (Integrated DNA Technologies; 10 pmol), BspE1 primer (Integrated DNA Technologies; 10 pmol) and 1 U *Taq*. PCR conditions were 20 cycles of 94°C for 10 sec, 49° for 30 sec, and 72°C for one min. Following the preselective amplification, a selective amplification was carried out to decrease the number of fragments amplified in each individual to approximately 200 by extending the primer on the 3' end. Preselective PCR products were diluted (1:10) and 2 μL of diluted product was amplified with primers (Table 1) to EcoRI+ AAG (Integrated DNA Technologies; 10 pmol) and BspEI +C (Integrated DNA Technologies; 10 pmol) with 1 U *Taq *in a 25 μL total volume. PCR conditions in the first cycle were 94°C for 10 sec, 65°C for 30 sec, and 72°C for one minute with the annealing temperature reduced by 0.5°C for 20 cycles, then 25 cycles of 94°C for 10 sec, 55°C for 30 sec, and 72°C for one minute.

**Figure 2:**
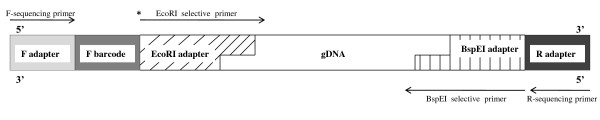
**Design of 454 pyrosequencing contig generated from the digestion of genomic DNA with restriction enzymes (EcoRI and BspEI), the addition of restriction site specific linkers, an individual barcode and a 454 amplicon adapter**.

Primers (Table 1) specific to the EcoRI restriction site were generated with the goals of labeling the DNA fragments from each individual with specific nucleotide barcodes [56] and preparing those samples for emulsion-based amplification. Starting at the 5' end, 19 nucleotides (Table 1) complementary to the primer on the DNA capture beads used in the emulsion PCR reaction [57] were synthesized (Integrated DNA Technologies). Following those nucleotides, each primer had a distinct 10 base pair barcode [56] used to identify individuals (ten primers in total). The final 19 base pairs of the primer were specific to the EcoRI adapter. The BspE1 primer (Table 1) started at its 5' end with 19 nucleotides (Table 1), which were complementary to the primer on the DNA capture beads followed by 18 base pairs specific to the BspE1 adapter (Figure 2). All primers were HPLC purified. Amplified selective fragments were diluted (1:10) and added to both EcoRI and BspE1 primers (Integrated DNA Technologies; 10 pmol) in a 25 μL volume. PCR conditions were 94°C for 10 sec, 50°C for 30 sec, and 72°C for one minute and were carried out for 30 cycles. PCR reactions were pooled into eight wells, where each of the ten distinct barcodes was represented only once in each of the pools. Each pool of PCR products was purified using QIAquick PCR Purification Kit (Qiagen, USA). PCR products were further purified with AMPure (Agencourt).

Emulsion PCR was carried out on PCR products as described [57]. Amplification of the PCR product on the bead was controlled for by quantifying and calculating the size of the amplicon pool using a Bioanalyzer 2100 so that there was a minimum of 2 × 10^6 ^copies of DNA that ranged in size from 100 to 700 base pairs. Subsequent products were sequenced on a Roche/454 Life Sciences GS FLX Sequencer at the University of South Carolina's Environmental Genomics Core Facility. The PicoTiter plate was subdivided into eight regions with an expectation of 30,000 reads per region [58].

### Assembly of pyrosequencing sequences and SNP Detection

Sequences were trimmed of their barcodes. All 626 sequences with at least one ambiguous base were removed since the presence of even a single ambiguous base is an effective indicator of low-quality sequence [59]. Because shorter than expected read lengths also correlate strongly with incorrect reads [60], another three percent of the sequences (whose lengths were smaller than 100 bp) were removed. The remaining reads were aligned using CAP3 [61]. Quality scores were rescaled to be comparable to the usual Phred Score using ARACHNE [62].

SNPs were called at both the individual level and population level. At the individual level, SNPs were called using both a Bayesian method and a likelihood ratio test (LRT) method. For the Bayesian method, 10^-4 ^was used as the prior for the mutation rate [63]. At the population level, for each locus on the contig, we simulated the error model and marked a locus as a potential SNP if it had a larger number of second alleles in comparison to the critical value from the error model. Furthermore, a potential SNP site had to have at least three individuals sequenced to 2× at that locus unless another potential SNP site was within five basepairs or over 90% of the individuals had been classified as heterozygous at the individual level. This was done to minimize the rate of false positives caused by homologs.

#### Bayesian and LRT model for SNP calling at individual level

For the Bayesian model, for each contig, *Prior *= 1 × 10^-4 ^represents the mutation rate; *N *represents the total number of unique mapping loci with multiple allelic types; *A*^*i *^and *a*^*i *^represent, respectively, the major and minor alleles at locus *i*; *N*_*i *_represents the total number of alleles observed for locus i, and *Y*_*j *_is the type of the *j*^*th *^allele copy among these *N*_*i *_alleles where *j *= 0 ⋯ *N*_*i*_; finally, *e*_*j *_is the probability of error of the *j*^*th *^allele where the error probability is computed as  and where *Q *is the corresponding quality score after rescaling.

The posterior probability for the *i*^*th *^locus being homozygous or heterozygous is:

Based on the posterior probabilities from above, we classified each of these N loci as homozygous or heterozygous exclusively. If a locus was classified as heterozygous, it was further tested using a likelihood ratio test (LRT) as follows:

For a particular locus *i *on the contig:

where *X*_*j *_stands for the true allele that we should have observed. For each *Y*_*j*_, we have an error probability of *e*_*j *_associated with it.

Then we have:

Therefore we have:

and

Based on all of the above, the likelihood of locus *I *was computed as:

Where *I*_*j *_= 1 if *Y*_*j *_= *A*_*i*_; and *I*_*j *_= 0 if *Y*_*j *_= *a*_*i*_

The LRT was performed with the hypothesis of *H*_*O*_: *p *= 0.5 *versus H*_*a*_: *p *>0.5 and -2 × *LRT *~ χ^2^(1).

#### Error model simulating

In order to call SNPs at the population level, we simulated the error model for each locus with multiple allelic types; we assumed that a particular locus was homozygous with major allele *A*^*i *^and randomly simulated *N*_*i *_number of alleles copies to be *A*^*i *^or any of the other three allele types from a uniform distribution with probability (1 - *e*_*j*_) and *e*_*j *_respectively. We repeated this process 10,000 times and recorded the different numbers of second alleles found in the simulation. The critical value was chosen as the number of second alleles with a right-side p-value of 0.001.

### Validation of SNPs

Multiplex assays targeting 458 SNPs in 250 *F*. *heteroclitus *individuals, 90 *F. grandis *individuals, 23 *F. majalis *individuals, and 21 *F. similis *individuals were attempted using the Sequenom MassARRAY technology. These consisted of 81 putative SNPs identified by the *F. heteroclitus *pyrosequencing, 350 putative SNPs previously identified in *F. heteroclitus *ESTs [64], and 27 putative SNPs from 22 genes containing, amongst others, SNPs in the aryl hydrocarbon receptor [65], lactate dehydrogenase B [29], and the proximal promoter of cytochrome P4501A (unpublished). Assays were designed using the MassARRAY Assay Design Software with the goal of maximizing multiplexing of 36 SNPs per well (Sequenom, San Diego, CA, USA). Only SNPs where 70 base pairs were annotated on either side of the polymorphism were included in the study. There were 14 SNPs previously identified with 454 pyrosequencing where this criterion was not met. If multiple SNPs were proximal (< 70 base pairs) to one another, one SNP was chosen and the other(s) was translated into a degenerate nucleotide (*e.g*., K = G or T). Reaction conditions were performed by iPLEX chemistry as recommended by Sequenom across 13 plates at the University of Minnesota's BioMedical Genomics Center. SNP genotypes were called using the Sequenom System Typer 4.0 Analysis package. This software uses a three-parameter model to calculate the significance of each putative genotype. Based on the relative significance, a final genotype is called and assigned a particular name (*e.g*., conservative, moderate, aggressive, user call). Non-calls also were noted (*e.g*., low probability, bad spectrum).

### Analysis of Genotype Data

Arlequin v.3.11 was used to calculate genetic diversity among populations (of *F. heteroclitus *and *F. grandis*) by calculating the percentage of polymorphic SNPs (*P*_*O*_), observed (*H*_*O*_) and expected heterozygosity (*H*_*E*_), and the within-population fixation index (*F*) [66]. Fixation index deviations from zero were tested by 10,000 permutations of alleles between individuals. Hardy-Weinberg equilibrium also was tested in each population. An analysis of molecular variance (AMOVA) was performed to calculate the distribution of variance within populations, between North and South regions, and between *F. heteroclitus *populations within North and South regions. For *F. grandis*, the AMOVA was performed to calculate the distribution of variance within populations as well as between populations longitudinally along the Gulf of Mexico. Since SNPs were initially identified from *F. heteroclitus *sequence data, a maximum of 5% missing data was used as a parameter for calculations involving *F. heteroclitus *and 10% for all others.

A Mantel test was performed to assess the assumption of isolation by distance using XLSTAT 2009 for *F. heteroclitus *and *F. grandis*.

STRUCTURE v.2.2 [67,68] was used to estimate the number of populations (K) in *F. heteroclitus*, *F. grandis*, *F. majalis *and *F. similis *along both the Western Atlantic and the Gulf of Mexico and to assign individuals to these populations. The Monte Carlo Markov Chain was run for 10^5 ^iterations following a burn-in period of 10^5 ^iterations for K = 1 to 14 using the correlated allele frequencies model and assumed admixture. Distruct v. 1.1 [69] was used to generate bar plots to depict classifications with the highest probability under the model. JMP Genomics 3.2 for SAS 9.1.3 conducted principal component analysis on all samples to establish population structure.

## Results

### GS FLX Sequencing and Assembly

A total of 111,001 reads were obtained in one run of the GS FLX instrument producing 5,346,445 total bases of sequence (average read length of 218 bases) with 99.98% of bases having a quality score of 20 or greater. Across the eight regions of the plate, there were on average 1,982 reads per individual. The third barcode produced many less reads per region (<1,000) amongst all regions. All other barcodes performed very similarly with respect to the number of reads per individual across regions. Only 46% of the number of expected reads (111,001 instead of 240,000) were obtained from sequencing. Prior to sequencing, the amplification success of loci on the beads was checked for quality using a Bioanalyzer 2100, and all samples passed. However, three of the eight regions produced half the expected number of reads and a fourth region produced only 15% the expected number of reads. This indicated local problems in sequencing with respect to particular regions and the samples in those regions rather than the plate as a whole. All control beads passed the filter control with an average percentage of 90% across all regions, whereas the percentage of samples passing the filter control varied between regions and averaged 36%: regions with fewer than expected reads had fewer samples passing the filter control. Two regions had very high failure rates due to mixed samples, indicating more than one amplicon per bead.

**Figure 3:**
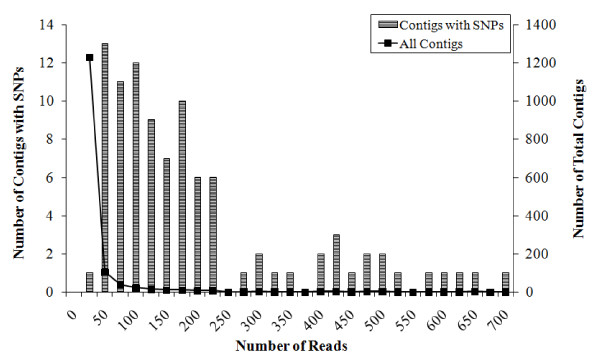
**Contig totals *versus *number of reads per contig amongst those contigs with identified SNPs (bars) and all contigs (squares)**.

Upon alignment 1,464 contigs were obtained with an average length of 213 base pairs. The average coverage across all loci was 22 reads per contig (Figure 3). Due to the low coverage of any one contig per individual, the detection of a SNP within a contig was mainly based on its presence across populations rather than at the individual level. Of the 1,464 contigs obtained, 96 contained SNPs. Within these contigs, 261 SNPs were identified. Among those contigs containing SNPs, the average length was 243 base pairs with an average coverage of 184 reads per contig (Figure 3). The observed rate of SNP detection is a function of depth, so as read counts per contig increased so did the number of SNPs detected. One third of all contigs with identified SNPs had only one SNP and 57% had two or fewer SNPs per contig. SNPs were distributed approximately evenly along the position in the contig (R^2 ^= 0.01).

### Genotyping success

Of the initial 458 loci we attempted to amplify, 277 had a greater than 90% successful call rate among all individuals with no more than two alleles per SNP. In *F. heteroclitus *61.4% of all loci amplified in greater than 95% of individuals. In *F. grandis*, 25.6% of SNPs did not amplify, and 58.2% of SNPs were monomorphic (Table 2). 24% of the monomorphic SNPs in *F. grandis *also were monomorphic in *F. heteroclitus*, but for the alternative allele, indicating fixed differences between the two species.

**Table 2:** Genotyping success of SNP markers using the MASSARRAY multiplex assay.

Category	Number of SNPs	Percentage of SNPs
SNPs called in >95% of *F. heteroclitus *individuals	259	61.4
SNPs called in <80% of all individuals	135	31.9
SNPs called in >90% but <95% of all individuals	101	23.9
Monomorphic SNPs called in >95% of all individuals	23	5.4
Polymorphic SNPs called in >95% of all individuals	163	38.6
SNPs called in <90% of all individuals identified in 454	35	43.2
SNPs called in >90% of all individuals identified in 454	46	56.8

**Figure 4:**
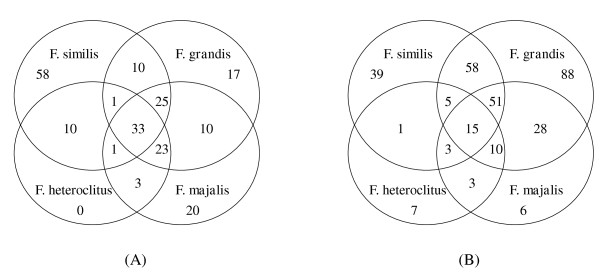
**Non-amplified and non-polymorphic loci among *Fundulus *species**. (A) Numbers of loci, which did not amplify with the MassARRAY platform among the four species of *Fundulus*. Not shown: loci shared between *F. majalis *and *F. similis *(8) and *F. heteroclitus *and *F. grandis *(12). (B) Numbers of loci, which were not polymorphic among the four species. Not shown: loci shared between *F. majalis *and *F. similis *(9) and *F. heteroclitus *and *F. grandis *(1).

On average, 80% (SD = ± 7.4%) of the putative SNPs identified with 454 pyrosequencing were amplified with MassARRAY in *F. heteroclitus *individuals: 72 of the 81 loci (88.8%) were polymorphic, 8 loci (9.8%) were monomorphic, and one locus did not amplify. Among all other putative SNPs genotyped with MassARRAY, 83% were successfully amplified. However, 13.5% of all loci in *F. heteroclitus*, 25.6% in *F. grandis*, 27.1% in *F. majalis *and 39.3% in *F. similis *did not amplify (Figure 4a). Many non-*heteroclitus *loci were also not polymorphic, and in *F. heteroclitus *12.3% of all loci were monomorphic, as were 58.2% in *F. grandis*, 26.4% in *F. majalis*, and 29.7% in *F. similis *(Figure 4b). Due to the divergence between species resulting in unsuccessful amplification in non-*heteroclitus *individuals, locus amplification success was addressed on a species and population level for all remaining tests and not on the overall amplification success. Due to the low sample size, amplification rate, and predominant monomorphism of loci in *F. majalis *and *F. similis *samples, further characterizations of genetic parameters (with the exception of population structure) were not carried out for these two species.

SNPs which were identified by Sequenom software as low probability in greater than 50% of all individuals were removed (17 SNPs in total). An additional 20 SNPs were excluded from analyses due to their excessive heterozygosity across individuals and populations of *F. heteroclitus*. These SNPs may represent segmental duplication where the two duplicate regions are identical, except that a SNP has been driven to high frequency or become fixed in one of the duplicates.

### Genetic Diversity

The percentage of polymorphic SNPs (*P*_*O*_) ranged from 3.7% to 67% (Table 3) among populations and species. The percentages of polymorphic SNPs were significantly different between northern and southern populations of *F. heteroclitus *where levels decreased in populations further north and east (p = 0.035). Among *F. grandis *populations, the percentages of polymorphic SNPs did not significantly differ along latitude (p = 0.143) or longitude (p = 0.415). Among populations, most loci were in Hardy-Weinberg equilibrium (Table 3). Observed heterozygosity (*H*_*O*_) among all populations ranged from 0.016 to 0.17 with a mean of 0.10 (Table 3). Observed heterozygosity was lower in northern *F. heteroclitus *in comparison to southern populations (p = 0.04) and did not differ along latitude (p = 0.72) or longitude (p = 0.33) in *F. grandis*. Average expected heterozygosity (*H*_*E*_) ranged from 0.019 to 0.20 with a mean of 0.11 (Table 3). The average within-population fixation index, *F*, averaged over all polymorphic loci was on average 0.16 in *F. heteroclitus *and 0.20 in *F. grandis *(Table 3).

**Table 3:** Genetic parameters of sampled populations in two species of *Fundulus*.

*Fundulus heteroclitus*					
**Population**	**P_O_**	**H_O_**	**H_E_**	**F**	**% Departure from HWE**

Maine	33	0.08	0.09	0.13†	7.0
Sandwich	48	0.12	0.14	0.13†	9.3
New Bedford Harbor	57	0.13	0.15	0.12†	7.9
Point Judith	44	0.11	0.13	0.18†	10.8
Clinton	59	0.12	0.13	0.09†	6.0
Newark	65	0.17	0.19	0.11†	6.4
Tuckerton	67	0.16	0.21	0.25†	12.5
Magotha	66	0.17	0.20	0.17†	11.5
Elizabeth River	67	0.16	0.20	0.23†	12.3
Manteo	65	0.16	0.20	0.19†	12.9
Georgia	51	0.13	0.16	0.19†	13.2

**Mean**	56.54	0.14	0.16	0.16	9.98
**Standard Deviation**	11.28	0.03	0.04	0.05	2.76

***Fundulus grandis***					

**Population**	**P_O_**	**H_O_**	**H_E_**	**F**	**% Departure from HWE**

Weeks Bay	9.0	0.016	0.019	0.13†	1.1
Dauphin Island	5.9	0.016	0.024	0.23†	2.8
Leeville	5.9	0.017	0.020	0.32†	2.0
Hackberry	10.1	0.023	0.032	0.10	3.0
Port O'Connor	8.5	0.018	0.026	0.27†	4.2
Port Aransas	3.7	0.021	0.031	0.23†	2.1

**Mean**	7.18	0.02	0.03	0.20	2.53
**Standard Deviation**	2.4	0.003	0.005	0.11	1.06

† p ≤ 0.01 based on 10,000 permutations between individuals within the same populations.

SNPs identified *via *454 sequencing did not have genetic parameters that differed from SNPs identified in ESTs with the exception of Hardy-Weinberg equilibrium. 454-derived SNPs had a higher percentage of SNPs not in Hardy-Weinberg equilibrium due to a lack of heterozygosity (22% *versus *9%).

Many SNP loci (60%) in *F. heteroclitus *had a frequency greater than 0.10 and were considered common SNPs (Additional file 1). In contrast, 90% of SNPs in *F. grandis *had low minor allele frequencies below 0.10.

### Population Structure

**Figure 5:**
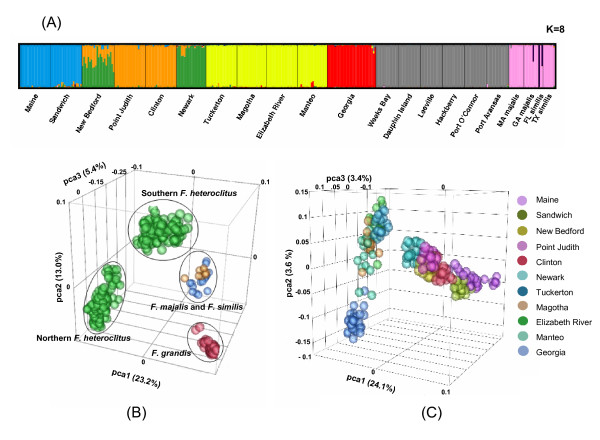
**Structure of *Fundulus *populations**. **(A)** Population structure as assessed by STRUCTURE. Bar plot was generated by DISTRUCT and depicts the classifications of the populations with the highest probability under the model. K indicates the number of clusters that maximized the probability of the model. Each individual is shown as a vertical bar. **(B) **Principal components PC1, PC2 and PC3 from all SNPs (as calculated in JMP Genomics 3.2) among all individuals. Species are separated from each other as well as northern and southern *F. heteroclitus *populations. Colors represent different species. **(C) **Principal components PC1, PC2, and PC3 from all SNPs among *F. heteroclitus *individuals. Colors represent different populations.

The two independent tests of population stratification (STRUCTURE and principle component analysis (PCA)) identified species and population differences in all samples (Figure 5). STRUCTURE analysis, which uses a Bayesian MCMC clustering approach to assign individuals to clusters, separated populations into eight different clusters (Pr(K) = 0.37; Figure 4a). At the most probable clustering of the data (K = 8), ten runs produced nearly identical membership coefficients which had pairwise similarity coefficients greater than 0.98. *F. heteroclitus *clustered north to south and *F. grandis *as its own separate cluster. Among *F. heteroclitus*, individuals from Maine and Georgia, the most northern and southern collection sites, formed their own distinct clusters. Individuals from sites between Maine and Georgia clustered with others from geographically similar sites. *F. majalis *and *F. similis *clustered together and away from the other two species. Similarly, in the PCA analysis, which does not rely on modeling the data, northern and southern *F. heteroclitus *stratified by latitude and were distinct from each other (Figure 5b) and each *F. heteroclitus *population was clustered together (Figure 5c). *F. grandis *made its own cluster and *F. majalis *and *F. similis *clustered together apart from other species (Figure 5b).

In *F. heteroclitus*, AMOVA showed that most of the variation was distributed within populations (59.05%), but another large proportion of variation (31.1%) was distributed among northern and southern regions. The remaining 9.85% of variation was explained by differences among populations within regions. In *F. grandis*, most of the variation was distributed within populations (82.4%), and a smaller proportion (17.6%) of variation was distributed longitudinally between populations across the Gulf of Mexico.

A Mantel test showed significant isolation by distance among *F. heteroclitus *populations (p < 0.001) and *F. grandis *populations (p = 0.032).

## Discussion

We used high throughput sequencing and genotyping technology to identify and verify SNP markers in four non-model species within the *Fundulus *genus. Genotype data sharply differentiated northern and southern populations of *F. heteroclitus *as well as other species in this genus (*F. grandis*, *F. majalis*, and *F. similis*). Within the species where SNPs were originally annotated, most can be successfully verified and used to study population structure as well as the role and outcome of selection forces on a genome-wide scale.

Using the 454 FLX pyrosequencing system, we observed 111,001 reads yielding an average of 22× coverage across 1,464 contigs. Read lengths and quality scores were similar to many other studies using the 454 FLX system to sequence uncharacterized genomes [8,70], but we identified fewer SNPs. Two-hundred and sixty-one SNPs were identified in 96 of these contigs (81 were further verified with the Sequenom MassARRAY platform). The percentage of contigs containing SNPs did differ between experiments: we obtained 0.07% of contigs containing SNPs while pyrosequencing of *Eucalyptus *ESTs identified 0.05% of contigs containing SNPs [8] and pyrosequencing of size selected, genomic DNA from swine identified 11.4% of contigs contained SNPs [70].

Our 454 pyrosequencing of genomic DNA was originally designed to both discover and genotype SNPs within and among populations of *F. heteroclitus*. Thus, we attempted to perform genome reduction with selective PCR reactions to approximately 200 loci that could be sequenced in 10 populations of 8 individuals. With 30,000 reads per one-eighth of a 454 sequencing plate, each region would have 15× coverage per individual or 980× coverage across all populations, enabling accurate genotype calls for most individuals. However, preselective amplication was not perfect, and many more than 200 loci were sequenced; most amplified only a single time in a single individual (these singlets therefore were not useful for variant detection). Furthermore, we obtained only 46% of the expected number of reads. In the end, these problems led to the inability to directly call individual genotypes. We were hoping to both identify SNPs and genotype individuals in a single step, but a more successful approach (as evidenced by the swine group [70]) is to make reduced representation libraries from many pooled individuals for SNP discovery followed by individual genotyping. Because a pool of individuals is used, this approach identifies few singlets and thus enhances the number of reads per contig. Furthermore, improvements in both the number and length of reads using the Titanium series FLX 454 system compared to the original FLX system we used will increase the number of identified SNPs.

To increase our ability to measure population genetic parameters within and among populations, we verified SNPs identified through 454 sequencing and additional SNPs annotated from *F. heteroclitus *cDNAs using the MassARRAY system. Similar percentages of 454 pyrosequencing derived SNPs and SNPs identified from ESTs were verified (80% and 83%, respectively). Of the 458 putative SNPs, 379 (82.75%) were polymorphic, but only 264 had a greater than 90% successful call rate among all individuals. Among *F. heteroclitus*, most SNPs amplified (61.3% were called in >95% of individuals) indicating that differences in amplification rate between species led to the lower overall call rate. In white spruce, 91% of SNPs verified with the Illumina SNP bead array platform [71,72] were true. Comparable to *F. heteroclitus*, 70% of SNPs in spruce were called in greater than 95% of individuals [52]. Overall, verification of SNPs was powerful in providing information over many markers and individuals and was able to provide data to determine differences within populations, between populations and between species.

Species differentiation was demonstrated using principle component analysis (PCA) as well as STRUCTURE analysis. Both analyses showed separation between *F. heteroclitus*, *F. grandis *and *F. majalis *and *similis *as well as population structure within *F. heteroclitus *(Figure 4). These analyses provided the most resolution (even among distinguishing populations) in *F. heteroclitus *because the SNPs were originally identified in this species (*i.e*., due to an ascertainment bias). PCA and STRUCTURE did not differentiate sister species, *F. similis *and *F. majalis*, from each other or establish population structure within these species. Small sample sizes (1 to 10 individuals per population), high levels of monomorphism (average of 28% of all SNPs), and the fact that only 10% of SNP alleles differed between these two species, decreased the power to detect such differences when analyzed in conjunction with *F. heteroclitus *and *F. grandis*. Population structure also was masked in *F. grandis *when data was analyzed with other species. However, when *F. grandis *individuals were analyzed separately, they also showed distinct population structure (data not shown). One other study has reported multiple fixed differences in mitochondrial sequences between *F. heteroclitus *and *F. grandis *[33], but no other study to date has evaluated differences at many loci between all four species used in this study.

Within *F. heteroclitus *and *F. grandis *species, within-population fixation indices (F_IS_, averaged across all loci) ranged from 0.09 to 0.32. Among *F. heteroclitus*, all populations had an overall significant deficiency of heterozygotes indicated by positive F_IS _values. In these populations, approximately 10% of loci had similarly very large F_IS _values (>0.5) across populations causing the skew in the average F_IS _value for each population. Within a population, these loci were predominately homozygous for one allele with a complete absence of the heterozygote and one or a few individuals homozygous for the alternative allele. The loci which presented this pattern were called conservatively at both alleles by Sequenom software across all individuals indicating that genotyping error was not the main reason for this pattern.

Furthermore, all northern populations were predominately homozygous for one allele and all southern populations were predominately homozygous for the alternative allele indicating strong demographic patterns in the data. The same demographic pattern was not found in *F. grandis*. Among *F. grandis *populations, most (70%) SNPs with high F_IS _values were different between populations. This is in contrast to *F. heteroclitus *populations where loci with high F_IS _values were shared across populations. Within any one *F. grandis *population, one allele was predominant as a homozygote with one or a few individuals with the alternative homozygote. The most parsimonious explanation is that there is undetected substructure.

SNPs in Hardy-Weinberg were shown to be moderately polymorphic (average of 60%) in *F. heteroclitus*. In *F. grandis*, SNPs were shown to lack polymorphism (7.18%). The higher percentage of monomorphic loci in *F. grandis *likely is due to ascertainment bias in SNP discovery caused by only using *F. heteroclitus *populations. Many of the monomorphic loci (24%) represent fixed differences between *F. heteroclitus *and *F. grandis*. Thus, while SNP markers developed in *F. heteroclitus *are not necessarily polymorphic in other *Fundulus *species, they still can be used to differentiate *F. heteroclitus *from other species.

Among *F. heteroclitus *populations, genotype data revealed strong latitudinal clines between the Northern and Southern *F. heteroclitus *populations. PCA, STRUCTURE, F_ST _values, and the isolation by distance test identified that individuals from Northern populations (above 40-41°N) were distinct from Southern populations. This split is centered around the southern-most extent of the Atlantic coastal advancement during the late Pleistocene [73]. Specifically, observed heterozygosity and allelic richness across all loci is significantly lower (p = 0.043, p = 0.042, respectively) in the north than in the south. These differences have been shown previously in morphological features [74] numerous allozyme loci [34-36,75] and microsatellites [28]. The larger historical population size of *F. heteroclitus *in the south [28] would maintain greater heterozygosity and allelic richness at shared loci; in the north, where population sizes are smaller, loci have a higher probability of becoming fixed.

Four STRUCTURE clusters encompass the six northern populations while only two clusters encompass the five southern populations (Figure 5A). Separate northern clusters may be driven by smaller population sizes in which drift is greater. When genetic drift has a larger effect it becomes easier to distinguish populations because the average difference in allele frequencies of a marker in different populations will be greater. This is illustrated by a larger average F_ST _of 0.20 among northern populations in comparison to that of an average F_ST _of 0.10 among southern populations. This statistic is also evident for the north and south split, where populations from respective regions had an extremely high F_ST _value of 0.44 when compared against one another. Similar genetic divergence has been reported for *F. heteroclitus *using microsatellites (0.196 among northern populations, 0.117 among southern populations and 0.330 for the two most divergent populations, Nova Scotia and Georgia [28]). Similar demographic patterns have been described in freshwater fish [76] and marine species such as goby [77] and blue crab [78], and, as in *Fundulus*, these patterns are attributed to Pleistocene events.

A similar latitudinal cline occurs between populations of *F. grandis*, and a Mantel test shows significant isolation by distance. However, there were no significant differences between either levels of polymorphism or observed heterozygosity along latitude or longitude. Williams *et al*., 2008 reported significant isolation by distance as well as decreased allelic richness with increasing latitude. In this 2008 study, microsatellites were used, and two additional sites southern to those used in our study were included. Since microsatellites have many more alleles than SNPs and two additional sites were found to have relatively higher allelic richness in comparison to all other sampling sites along the gulf, this may account for the differences found in levels of polymorphism.

## Conclusions

By targeting SNPs contained in both coding and non-coding areas of the genome, we are able to better understand how evolutionary forces are shaping the *Fundulus *genome. Similar studies using high throughput methods to sequence SNP markers have been developed in Atlantic cod [51], white spruce [52], *Eucalyptus *[8], and swine [70]. Like our study, these studies expanded their own species' knowledge base with respect to potential markers for studying evolutionary adaptation (in the case of cod and spruce), genome-wide assessment of diversity (*Eucalyptus*) or for use in breeding programs (swine)

## Authors' contributions

LMW designed experiments, carried out laboratory and statistical analyses, and drafted the manuscript. XM carried out SNP detection of 454 data. ARB carried out SNP detection of 454 data, assisted in statistical analyses of MassARRAY data, and provided comments on earlier versions of this manuscript. CDB assisted in designed experiments and help develop SNP detection software. MFO designed experiments, assisted on statistical analysis, and helped to draft the manuscript. All authors read and approved the final manuscript

## Supplementary Material

Additional file 1 **SNP minor allele frequencies**. Distributions of SNP minor allele frequencies (MAF) within *F. heteroclitus *and *F. grandis *populations. Click here for file
