# Supplementary material for: SNP identification, verification, and utility for population genetics in a non-model genus
Source: BMC Genet. 2010 Apr 30;11:32. doi: 10.1186/1471-2156-11-32 (PMC2874759; doi:10.1186/1471-2156-11-32)
Supplement: Additional file 1 — SNP minor allele frequencies. Distributions of SNP minor allele frequencies (MAF) within F. heteroclitus and F. grandis populations. [file 1471-2156-11-32-S1.PDF]

Additional File 1. Distributions of SNP minor allele frequencies (MAF) within *F. heteroclitus* and *F. grandis* populations.

		**Average MAF**							
	# SNP	Including Monomorphic	Excluding Monomorphic	Monomorphic	0≤MAF≤0.1	0.1≤MAF≤0.2	0.2≤MAF≤0.3	0.3≤MAF≤0.4	0.4≤MAF≤0.5
F. heteroclitus	398	0.2	0.25	0.21	0.19	0.11	0.16	0.15	0.18
F. grandis	410	0.03	0.13	0.74	0.16	0.03	0.02	0.02	0.03
**Overall**	404	0.12	0.19	0.48	0.18	0.07	0.09	0.09	0.11
